# A mental health first aid training program for Australian Aboriginal and Torres Strait Islander peoples: description and initial evaluation

**DOI:** 10.1186/1752-4458-3-10

**Published:** 2009-06-03

**Authors:** Len G Kanowski, Anthony F Jorm, Laura M Hart

**Affiliations:** 1 Mental Health First Aid Training and Research Program, ORYGEN Youth Health Research Centre, University of Melbourne, Parkville, Victoria 3052, Australia

## Abstract

**Background:**

Mental Health First Aid (MHFA) training was developed in Australia to teach members of the public how to give initial help to someone developing a mental health problem or in a mental health crisis situation. However, this type of training requires adaptation for specific cultural groups in the community. This paper describes the adaptation of the program to create an Australian Aboriginal and Torres Strait Islander Mental Health First Aid (AMHFA) course and presents an initial evaluation of its uptake and acceptability.

**Methods:**

To evaluate the program, two types of data were collected: (1) quantitative data on uptake of the course (number of Instructors trained and courses subsequently run by these Instructors); (2) qualitative data on strengths, weaknesses and recommendations for the future derived from interviews with program staff and focus groups with Instructors and community participants.

**Results:**

199 Aboriginal people were trained as Instructors in a five day Instructor Training Course. With sufficient time following training, the majority of these Instructors subsequently ran 14-hour AMHFA courses for Aboriginal people in their community. Instructors were more likely to run courses if they had prior teaching experience and if there was post-course contact with one of the Trainers of Instructors. Analysis of qualitative data indicated that the Instructor Training Course and the AMHFA course are culturally appropriate, empowering for Aboriginal people, and provided information that was seen as highly relevant and important in assisting Aboriginal people with a mental illness. There were a number of recommendations for improvements.

**Conclusion:**

The AMHFA program is culturally appropriate and acceptable to Aboriginal people. Further work is needed to refine the course and to evaluate its impact on help provided to Aboriginal people with mental health problems.

## Background

Mental health first aid has been defined as "the help provided to a person developing a mental health problem or in a mental health crisis. The first aid is given until appropriate professional treatment is received or until the crisis resolves" [1]. In order to increase the mental health first aid skills of the public, Kitchener and Jorm [2,3] developed a MHFA training course in Australia. This course has been found to improve knowledge, attitudes and helping behaviour in two randomized controlled trials with the Australian public [4]. The course is now widely disseminated in Australia and has spread to many other countries [5].

In developing this course, it was recognized that there is a significant cultural element to mental health first aid. Culturally-sensitive training of the public needs to take account of such factors as: the cultural group's dominant understanding of mental disorders, particular risk and protection factors operating in that group, cultural rules that may affect what are considered appropriate first aid actions, and the availability of culturally-appropriate professional help to which a person may be referred. For this reason, it has been necessary to carry out cultural adaptation of the course as it has spread to other countries and to accommodate the needs of cultural minorities within a country [5].

One cultural group within Australian society with particular mental health needs are Aboriginal and Torres Strait Islander peoples, who comprise 2.4% of the Australian population. Although this percentage is small, it comprises around 500,000 people, many of whom have high levels of physical illness, lower life expectancy, and high levels of psychological distress. Many live in socially disadvantaged environments, with limited access to transport, poor housing and low incomes [6-8].

Historically, Aboriginal people have suffered significant losses since Australia was colonised in the 1700s. These include loss of land, loss of traditional hunting grounds, loss of traditional language, forced relocation onto missions and reserves, loss of cultural and legal traditions, and the forced removal of children. These significant losses and social upheavals have impacted negatively on Aboriginal people, and have left many individuals, families and communities significantly traumatised and grief stricken. This has resulted in large numbers of people being at high risk for developing a range of mental health problems and mental illness. Current data indicates high levels of self-reported psychological distress, with symptoms of anxiety and depression twice as common in Aboriginal people compared to non-Aboriginal people [6], higher rates of hospitalisation for intentional self harm, and higher suicide rates compared to non-Aboriginal Australians [7,8]. Although mental illnesses are prevalent, and suicide rates unacceptably high, most Aboriginal communities receive little education on how to manage mental health problems or to deal with mental health crises.

The *'Ways Forward' National Consultancy Report on Aboriginal and Torres Strait Islander Mental Health *[9] advocated the use of strategies that provide general community education programs concerning the recognition, responses and prevention of suicidal behaviours and mental health problems. Similar recommendations were identified by the *National Strategic Framework for Aboriginal and Torres Strait Islander Peoples' Mental Health and Social and Emotional Wellbeing 2004 – 2008 *[10] and the *National Mental Health Plan 2003–2008 *[11]. All of these documents called for increases in the levels of mental health awareness and increased levels of mental health literacy in the community, including Aboriginal communities. The adaptation of the MHFA program for Aboriginal and Torres Strait Islander peoples was seen as one means of fulfilling some of the recommendations made in these documents.

### Process of cultural adaptation

Consultations with Aboriginal and Torres Strait Islander individuals and groups between 2004 and 2006 indicated the need to modify the MHFA course developed by Kitchener and Jorm [3] to better suit the needs of Aboriginal and Torres Strait Islander peoples. To guide this adaptation, an AMHFA Working Group was established in 2006, comprising a small body of Aboriginal people with professional experience in the Aboriginal mental health field and Len Kanowski who is one of the MHFA Trainers of Instructors. An AMHFA Manual was finalised by MHFA staff in conjunction with the AMHFA Working Group by working through the existing Australian manual and course materials and commenting on where specific adaptations were needed. The changes were then made by Len Kanowski in conjunction with other MHFA staff. Materials that were culturally adapted for the AMHFA program included a manual [12], teaching notes, resource folder, PowerPoint slides, and DVDs featuring Aboriginal people talking about mental health problems or demonstrating mental health first aid skills. A workbook was also developed [13]. The final version of the materials was approved by the AMHFA Working Group. Culturally appropriate Aboriginal social and emotional well-being and mental health materials were also sourced and included in the instructor training kit. All these materials were deemed by the Working Group to be culturally appropriate and relevant to community needs before being included in the program. The Working Group was disbanded at the end of December 2006 once the materials were finalised to their satisfaction.

An important result of the cultural adaptation process was that the materials were empowering for Aboriginal people and acknowledged their resilience in surviving historical traumas and losses. Aboriginal artwork was used throughout the teaching materials and manual to illustrate important messages and Aboriginal concepts of mental health and well-being, and to give a clear identification of these materials as belonging to Aboriginal and Torres Strait Islander people.

The final adapted AMHFA package involved a 14-hour course for Aboriginal and Torres Strait Islander community members and a 5-day Instructor Training Course. It was anticipated at the beginning of the project that further adaptations would need to take place to meet the needs of Aboriginal communities with lower levels of written literacy, and that further adaptations of the course and materials would be informed by feedback from the AMHFA Instructors and the outcomes of the initial evaluation.

### The need for AMHFA Instructors

An important feature of the MHFA program in general has been its devolved organization. The MHFA Training and Research Program at Orygen Youth Health Research Centre does not employ Instructors. Rather, it works in partnership with other organizations working at the local level, who employ Instructors and sponsor courses. This means that Instructors are based in the community where they are working and that any skills they acquire through attending AMHFA Instructor training are more likely to stay in that community. This principle of local partnership is particularly important when working with and within Aboriginal and Torres Strait Islander communities.

During the process of adapting the course, staff at MHFA and the Office of Aboriginal and Torres Strait Islander Health (OATSIH) identified a need to increase the number of Aboriginal and Torres Strait Islander Instructors to conduct the culturally adapted AMHFA course in Aboriginal and Torres Strait Islander communities. At the time the cultural adaptation was carried out, there were only three Instructors who identified as Aboriginal. To increase the existing number of accredited AMHFA Instructors who would then conduct the 14-hour AMHFA course nationally, OATSIH provided scholarships to allow the training of additional Instructors in 2007–2008. Successful applicants were required to meet the following criteria prior to undertaking the 5-day AMHFA Instructor Training Course:

1. Aboriginal and/or Torres Strait Islander person;

2. Good knowledge of mental disorders and their treatment;

3. Personal or professional experience with people with mental health problems;

4. Good teaching and communication skills;

5. Good background knowledge of mental health and community services;

6. Good organisational support (as shown by a letter of support from the applicant's supervisor) to ensure AMHFA sustainability

Two Trainers of Instructors (Len Kanowski and Kara Eddington) conducted a series of 5-day Instructor Training Courses across Australia. Once Instructors had the initial training, the Trainers of Instructors continued to provide them with on-going support (face-to-face, telephone and e-mail) as they began to offer 14-hour AMHFA courses in their own communities.

### Approach to evaluation

This paper reports an initial evaluation of the AMHFA program. The evaluation can be usefully considered using the framework of Campbell et al. [14] for the design and evaluation of complex interventions to improve health. These authors propose that evaluation of complex interventions proceeds through four phases:

1. Modelling (identifying the components of the intervention which are likely to work);

2. Exploratory trial;

3. Definitive randomized controlled trial; and

4. Long-term implementation.

The present evaluation corresponds to Phase 1 of this framework. It provides a basis for improving and refining the intervention before a formal trial is carried out. After these improvements have been carried out, the AMHFA program will be ready for a Phase 2 exploratory trial.

## Methods

Quantitative data were collected to measure number of Instructors trained in the 5-day Instructor Training Course and number of 14-hour AMHFA courses subsequently run by these Instructors. We also examined predictors of whether Instructors subsequently ran courses. Qualitative data were collected to ascertain the perceptions of various stakeholders about the strengths, weaknesses and possible future directions of the AMHFA program.

### Measuring uptake of the Instructor Training Course and the 14-hour AMHFA course

Number of Instructors trained and courses run was ascertained using the Instructor Database which is associated with the MHFA website . After completing their training, Instructors are recorded on this database. The database then allows Instructors to record details of all courses they teach. To encourage entry of data, all AMHFA Instructors were contacted during the months of August to October 2008 by the AMHFA training team and assistance was provided if the Instructor had not entered information on all the courses they had taught. The data were then taken from the database on 1 November 2008, which was the census date for this component of the evaluation. The data were plotted to show the percentage of Instructors running a course as a function of time since training.

Some non-Aboriginal people also trained to be Instructors so that they could work as a team with an Aboriginal person to run courses in Aboriginal communities. Acceptance into this training program required participants to meet strict criteria, including previous experience as an accredited MHFA Instructor presenting the 12-hour standard version of the course, experience in working in the field of Aboriginal mental health, ongoing work within Aboriginal communities and a commitment to involving Aboriginal people as cultural experts within the 14-hour AMHFA courses they present in the future. Data on the number of these Instructors were also taken from the database.

### Analysis of predictors of conducting courses

Because many Instructors had not run courses by the census date, a statistical analysis was carried out to find characteristics of Instructors who had run one or more courses compared to those who had not. One obvious predictor is the time since training – the more months available to run a course, the greater the probability that an Instructor will do so. However, we were interested to find predictors additional to the time available. These predictors might give clues to better selection of Instructors or better support that could be provided post-training in the future. The following predictors were examined:

#### Instructor characteristics

1. Gender of Instructor

2. Type of employer (including whether it was a health or mental health related organization)

3. Type of position of employment (including whether it was a health or mental health worker role)

4. State in which the Instructor resided

5. Remoteness of the Instructor's home town according to the RRMA categorization of the Australian Bureau of Statistics

6. Number of years experience in mental health prior to Instructor training

7. Highest qualification achieved

8. Number of years experience in teaching or training prior to Instructor training

9. Did the Instructor identify as a consumer of mental health services for a mental illness?

10. Did the Instructor identify as a carer of a loved one with a mental illness?

11. Did the Instructor indicate on their application form that they would like to become an Instructor to improve their mental health knowledge?

12. Did the Instructor indicate on their application form a desire to teach, educate, present, deliver or train?

#### Post-course support provided

1. Had the Instructor had any form of contact with a Trainer of Instructors?

2. Had the Instructor had phone contact with a Trainer?

3. Had the Instructor had email contact with a Trainer?

4. Had the Instructor met face-to-face with a Trainer?

5. Had the Instructor attended an evaluation workshop?

6. Had the Instructor attended the annual Instructor Conference?

The predictors were examined using logistic regression analysis. The analysis was carried out in two steps. In the first step, each predictor was examined individually, with time since training as a covariate. In the second step, any predictor that was significant at the P < 0.10 level was entered into a simultaneous regression analysis, again with time as a covariate. Predictors significant at the P < 0.05 level were interpreted.

### Qualitative evaluation

Accredited AMHFA Instructors were invited to attend regional evaluation workshops. Instructors were asked to comment on their perceptions of both their 5-day Instructor Training Course as well as the structure and materials of the 14-hour AMHFA course they were to present in their communities. The workshops were conducted in New South Wales, Queensland, Northern Territory, and Western Australia. An Aboriginal Trainer of Instructors, who is also a psychologist, facilitated these forums, while a member of the evaluation team audio-recorded the proceedings and typed a summary of the points raised on a laptop. Immediately following each workshop, these two staff reviewed the points that were recorded on the laptop and reached a consensus that these were correct and that there were no omissions.

Several evaluation workshops were also offered to community members, and staff from Aboriginal community controlled health services, who had attended a 14-hour AMHFA course. Workshops were offered within specific Aboriginal community controlled health service sites. Participants were invited to attend the workshops by the Aboriginal Instructors who conducted the course. Workshops were held in New South Wales, Queensland, South Australia and Western Australia. These workshops were also facilitated and recorded as described above. The discussion dealt with how participants found their course, the information presented, and how confident they felt about providing mental health first aid to an Aboriginal or Torres Strait Islander person. Following each workshop, the information gathered was reviewed as described for the Instructor workshops.

The two Trainers of Instructors and the AMHFA administrative assistant were each interviewed by two members of the evaluation team; one who conducted the interview while the other member recorded the points made. The Trainers of Instructors were asked to talk about what they saw as the strengths and weakness of the program and suggestions for future improvements. For the administrative assistant, the interview focussed on administrative aspects only.

### Analysis of the qualitative data

There were a large number of points made across the various workshops and interviews. These were grouped into three broad categories by one of the evaluation team (LMH): (1) Perceived strengths, (2) Areas for improvement, and (3) Future directions. Some points emerged consistently across the different workshops and interviews, whereas others were idiosyncratic. In order to find the most consistently mentioned points, we selected out those that were mentioned in at least 3 of the 4 Instructor workshops, in at least 3 of the 4 course participant workshops, or by at least 2 of the 3 AMHFA training and administrative team. These consistently mentioned points formed the basis of conclusions reached about strengths, weaknesses and future directions.

### Ethics approval

Ethics approval was provided by the Melbourne Health Mental Health Research Ethics Committee. The committee carried out consultation with an expert in Aboriginal health research when making its decision.

## Results

### Uptake of the Instructor Training Program

The inaugural 5-day AMHFA Instructor Training Course commenced on 5^th ^March, 2007. By 1^st ^November 2008, 199 Aboriginal Instructors had been trained in 17 Instructor Training Courses, which were held across the country. In addition, the AMHFA Training Program had run two 3-day training courses for non-Indigenous, previously accredited, MHFA Instructors. At the census date, there were 8 accredited non-Indigenous AMHFA Instructors.

### Uptake of the 14-hour AMHFA courses

As of November 2008, 155 14-hour AMHFA courses had been run, with 1,115 people attending. The analyses presented here include the 14-hour courses that were run between June 25^th ^2007 and November 30^th ^2008. Of these 115 courses, 58 (50%) were presented in a co-facilitation format, where two or more Instructors shared the presenting, and 14 courses (12%) involved a co-facilitator who was a non-Aboriginal person. Four of those 14 courses involved a co-facilitator who had completed the 3-day AMHFA Instructor course, while the other 10 courses involved a co-facilitator who had completed the 5-day standard MHFA Instructor course only. There was one 14-hour AMHFA course that was presented by two non-Indigenous AMHFA accredited Instructors, with no Aboriginal Instructor present.

### Predictors of conducting courses

The analyses of predictors of conducting a course included only the 165 Instructors who were trained between March 2007 and the end of August 2008. More recently trained Instructors had not had sufficient time to have prepared for and presented a 14-hour AMHFA course in their communities.

**Figure 1:**
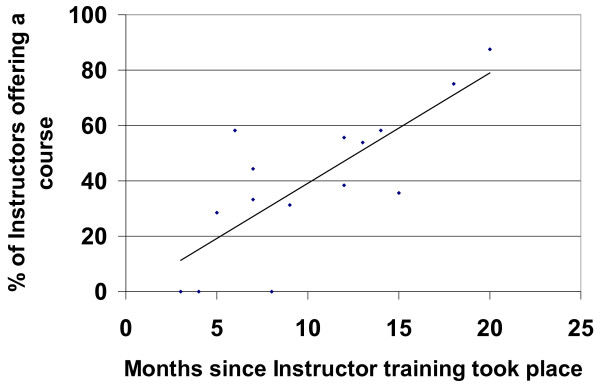
**Percentage of Instructors offering an AMHFA course following each of the 5-day Instructor Training Courses**. (A regression line has been fitted to the data points).

Of the 165 Instructors who were trained between March 2007 and August 2008, 67 (40%) had run one or more courses. The main factor associated with whether or not a course was taught was time since training. Figure 1 shows the percentage of Instructors who had run a course as a function of when their training took place. It can be seen that the percentage is quite high for those Instructors who attended the earliest Instructor Training Courses.

In order to assess if there were any characteristics of Instructors that may have predicted whether or not they presented a course, a series of logistic regression analyses was performed. The length of time Instructors had between completing their 5-day training and the census date was the most reliable predictor of whether or not an Instructor had run a course. For each week since the Instructor Training Course, the odds ratio was 1.04, P < 0.001.

A set of further analyses were performed, controlling for time since Instructor training, to see if any other variables could increase the likelihood that an Instructor would run a course. These further analyses found that the number of years of prior teaching or training experience significantly increased the likelihood of presenting a course (OR = 1.39, P = 0.017). The only other variable that predicted course presentation was whether or not an Instructor had had contact with a Trainer of Instructors (OR = 2.75, P = 0.007). In particular, having face-to-face contact with a Trainer was a strong predictor of course presentation (OR = 3.52, P = 0.001). All other predictors were non-significant, including whether the Instructor worked in a health-related role and the remoteness of where they worked.

While anecdotal evidence from Instructors during their contact with Trainers of Instructors suggested that the level of encouragement and support from their employing organisation was more likely to facilitate courses being conducted, the evaluation procedure was not able to directly quantify "organisational support", as it was a requirement of all organisations to provide a letter of support upon the Instructor's application to train. In order to overcome this difficulty, the type of employer (e.g. health, university, government) and the type of job the Instructor was employed in was evaluated (e.g. case manager, lecturer, community support officer). It was hypothesised that where Instructors were employed in positions that directly involved the delivery of AMHFA courses as part of their position description, such as Education Officer, they would be more likely to present a course. It is therefore interesting to note that neither type of employer nor position of employment reliably predicted the number of courses taught.

The set of predictors that were significant at the P < .10 level were next included in a simultaneous multiple logistic regression analysis. The only predictors which remained significant when all other predictors were adjusted for were time since training and the Instructor having had face-to-face post-training contact with one of the Trainers of Instructors.

### Qualitative findings

Eight focus groups were held in locations across Australia, with a total of 56 participants: 34 Instructors and 22 course participants. Instructors were asked to comment on their perceptions of both the Instructor Training Course, as well as the structure and materials of the AMHFA course they were to present in their communities. Course participants were asked to comment on how they found the course, the information presented, and how confident they felt about providing mental health first aid to an Aboriginal or Torres Strait Islander person. In addition, individual interviews were conducted with AMHFA staff to evaluate staff attitudes towards the program.

The overwhelming response from all Trainers of Instructors, Instructors and 14-hour course participants was that the 5-day Instructor Training Course and 14-hour AMHFA course are culturally appropriate, empowering for Indigenous people, and provided information that was seen as highly relevant and important in assisting Aboriginal people with a mental illness.

A number of suggestions for changing the course were put forward consistently:

1. The qualitative data supported the idea of spreading the Instructor training over more days with a break in between. One suggested way of implementing this idea was to require Instructors to do the 14-hour AMHFA course first before applying to become an Instructor. This would give potential Instructors a clearer idea of what an Instructor is expected to do. If there was a gap between the 14-hour course and the Instructor training week, it would also allow more time for them to absorb the material and space out the learning of the large amount of information that Instructors are presented with.

2. There was widespread support for the cultural appropriateness of the training approach and the training materials. Nevertheless, some areas were identified as needing strengthening. A particular need is more adaptations of the materials to suit less literate course participants and those who learn by doing rather than by reading. While the AMHFA workbook was developed with this in mind, it is evident that it has not met this need. One idea put forward was for a pictorial flip-chart. There were also suggestions for greater use of role playing. Other areas for improvement that were suggested were developing DVDs involving Aboriginal people, incorporating more Aboriginal statistics and incorporating more local information on services etc. The issue of DVDs has already been covered by the development of AMHFA films involving Aboriginal people, which were released in late 2008, after the census date.

3. Instructors mentioned that they needed post-training support initially to give them confidence to begin and that there were sometimes workplace barriers that had to be overcome. They also reported the need to debrief and to seek specific advice on issues that arose following teaching a course.

## Discussion

This initial evaluation has shown that it is possible to develop and implement a culturally-acceptable version of MHFA for Aboriginal and Torres Strait Islander peoples. There are a number of factors that may have contributed to the success of the course and program, including: (1) The MHFA course and manual developed by Kitchener and Jorm [2,3] provided a sound evidence-based platform on which to develop a AMFHA course; (2) The input of an Aboriginal reference group to guide the adaptation; (3) Indigenous staff members were employed within the AMHFA Training and Administration team to provide cultural input and on-going support to Instructors; (4) Instructor Training Courses were provided in a variety of locations to give national coverage, including in rural and remote areas; (5) An evaluation strategy to inform and guide the ongoing development and improvement of the AMHFA course and program was established prior to project commencement and resulted in many positive recommendations for course and program improvement.

Most Instructors were found to offer AMHFA courses if given sufficient time to do so. However, there are a significant number of Instructors who do not. Even if some Instructors never run a course, it is arguably still a good investment in workforce development because of the additional training that was received during the 5-day Instructor Training Course. Nevertheless, it would be better use of resources to focus the training on people who are more likely to deliver courses. We attempted to find predictors of Instructors who were successful in running a course in the follow-up period. We found some indication that successful Instructors had more prior teaching experience. However, the prediction was not strong enough to recommend any additional selection criteria than those already used. There were Instructors with a wide variation in backgrounds who were successful in running courses. It seems more sensible to make the Instructor training available as at present and let the best people self-select afterwards by demonstrating that they can run courses successfully.

The analysis of predictors of whether or not an Instructor offered a course found that post-training contact with AMHFA staff, particularly, face-to-face contact was associated with a greater likelihood of being a successful Instructor. Unfortunately, it is not possible to be certain what is cause and what is effect here. It is possible that post-training support helped the Instructors to run a course. It is also possible that Instructors who intended to run a course were more likely to make contact for support. Either way, it appears that Instructors wanted support.

We are aware that there may be other factors determining whether or not an Instructor ran a course that we were unable to measure. It is possible that factors within the Instructor's working environment and community are as important as the Instructor's own characteristics in determining whether courses are taught. For example, although we asked Instructors to provide a letter of support from their employer when they were accepted into the training program, it is likely that there was variable support and opportunity in the workplace to actually offer courses. Similarly, there may be important community pressures that affect the Instructor's ability to offer courses. Examples include family pressures, sorry business (community grieving), changing positions of employment and political factors such as the Federal Government's intervention to stop child abuse in some communities.

There was widespread support in the qualitative data for the cultural appropriateness of the training approach and the training materials. Nevertheless, some areas were identified as needing strengthening. A particular need is more adaptations of the materials to suit less literate course participants and those who learn by doing rather than by reading.

We also acknowledge that there is significant cultural diversity amongst Aboriginal and Torres Strait Islander communities and that a 'one size fits all' approach is not appropriate. We see this training program as needing adaptation at a local level by Instructors who know their community well, and there is a longer-term need to develop materials tailored to specific communities and groups. However, we had to make a start somewhere, and see the present course as a platform on which to build further approaches.

Another parallel development that is relevant to the next revision of the manual and materials is a project funded by the *beyondblue: the national depression initiative *to develop mental health first aid guidelines for Aboriginal people using consensus of Aboriginal mental health experts. This project has developed expert consensus guidelines on: *Cultural Considerations and Communication Techniques*, *Trauma and Loss*, *Suicidal Thoughts and Behaviours*, *Deliberate Self-Injury*, *Depression *and *Psychosis *[Hart LM, Jorm AF, Kanowski LG, Kelly CM, Langlands RL: Mental Health First Aid for Indigenous Australians: using Delphi consensus studies to develop guidelines for culturally appropriate responses to mental health problems: Submitted]. Additional guidelines are currently being developed on first aid for problem drinking and problem drug use. These guidelines give a strong basis guiding the content of the AMHFA course. However, because they have only gradually become available during the period of course development and rollout, they have yet to be incorporated.

### Future evaluation of the program

The next step needed is a Phase II exploratory trial. This would focus on measuring the impact of the training on the knowledge, attitudes and behaviour of 14-hour AMHFA course participants. The methods of measuring these outcomes need careful consideration to ensure that they are acceptable and culturally appropriate for Aboriginal people. Past research on the standard MHFA course has used written questionnaires. However, this method may not be appropriate for Aboriginal participants who vary in literacy and may prefer oral approaches to assessment. One possibility would be to use the method of systematically gathering and analysing stories of providing mental health first aid that was used for an evaluation of the standard MHFA course in a rural area [15]. Because stories are a familiar way of communicating for Aboriginal people, this method might be particularly appropriate.

## Conclusion

The evaluation of the AMHFA program indicates that the course adaptation and roll-out has been very successful on a number of fronts. The success of the program bodes well for future adaptations and the growth and development of the program. However, while the initial phase of this program appears very successful, it relies on Government support to continue the work beyond this phase. At the present time, it is uncertain whether funding will be available to sustain the program in the long-term.

## Competing interests

LGK is the principal developer of the AMHFA program.

## Authors' contributions

LGK coordinated the development of the program, secured funding, was an editor of the course manual and teaching materials, and co-wrote the paper. AFJ was an editor of the course manual, an investigator on the evaluation study and co-wrote the paper. LMH was an investigator on the evaluation study and co-wrote the paper. All authors read and approved the final manuscript.
